# Management of Inflammatory Arthritis in pregnancy: a National Cross-Sectional Survey of Canadian rheumatologists

**DOI:** 10.1186/s41927-019-0065-8

**Published:** 2019-05-17

**Authors:** Mary A. De Vera, Corisande Baldwin, Nicole W. Tsao, Alyssa Howren, Glen S. Hazlewood, Nevena Rebić, Stephanie Ensworth

**Affiliations:** 1 University of British Columbia, Faculty of Pharmaceutical Sciences, Vancouver, BC Canada; 2 Arthritis Research Centre of Canada, Richmond, BC Canada; 3 Collaboration for Outcomes Research and Evaluation, Vancouver, BC Canada; 4 Division of Rheumatology, Department of Medicine, Faculty of Medicine, University of British Columbia, Vancouver, BC Canada; 5 Cumming School of Medicine, University of Calgary, Calgary, AB Canada

**Keywords:** Pregnancy, Inflammatory arthritis, Rheumatic diseases, Survey

## Abstract

**Background:**

With improved therapies and management, more women with inflammatory arthritides (IA) are considering pregnancy. Our objective was to survey rheumatologists across Canada about their IA management in pregnancy to identify practice patterns and knowledge gaps.

**Methods:**

We administered an online survey with questions regarding medications for IA treatment including conventional synthetic disease modifying antirheumatic drugs (csDMARDs) and biologics/small molecules in planned and unplanned pregnancies. Email invitations were sent to members of the Canadian Rheumatology Association. We calculated responses frequencies and a priori set a cut-off of ≥75% to define consensus.

**Results:**

Ninety rheumatologists participated in the survey (20% participation rate); 57% have been practicing for > 10 years, 32% for ≤10 years, and 11% in training. There was consensus on discontinuation of 4 csDMARDs – cyclophosphamide (100%), leflunomide (98%), methotrexate (96%), and mycophenolate mofetil (89%) – in planned pregnancies but varied responses on when to discontinue them or what to do in unplanned pregnancies. Respondents agreed that 3 csDMARDs – azathioprine (84%), hydroxychloroquine (95%), and sulfasalazine (77%) – were safe to continue in planned and unplanned pregnancies. There was consensus with use of 4 biologics – adalimumab (81%), certolizumab (80%), etanercept (83%), and infliximab (76%) – in planned pregnancies but uncertainty on when they should be discontinued and their use in unplanned pregnancies.

**Conclusions:**

This national survey shows consensus among rheumatologists on the use of some csDMARDs and biologics/small molecules in IA patients planning pregnancy but varied knowledge on when to discontinue and what to do in unplanned pregnancies.

**Electronic supplementary material:**

The online version of this article (10.1186/s41927-019-0065-8) contains supplementary material, which is available to authorized users.

## Background

Inflammatory arthritides (IA), which include rheumatoid arthritis (RA) and systemic lupus erythematosus (SLE), are chronic autoimmune diseases that disproportionately affect females more than males [1], often with peak incidences during reproductive years [2]. Although, historically some IA were recognized to improve during pregnancy, particularly RA [3], recent evidence suggests remission during pregnancy in less than 20% of women with RA [4]. Consequently, it is estimated that 40–50% of women with IA require treatment throughout the perinatal period [5, 6].

Managing IA pregnancy is an important clinical challenge. A 2013 UK survey of rheumatologists and obstetricians showed no uniform practice for using IA medications during pregnancy. Although, no cut-off was used to define response consensus, 80% of respondents indicated continuation of hydroxychloroquine and over 98% advised discontinuation of methotrexate and leflunomide [7]. Additionally, over 92% of respondents indicated discontinuation of anti-tumour necrosis factor agents (anti-TNFs) and rituximab [7]. Since practice patterns differ across health care systems, these findings may not be generalizable. Guidelines for perinatal use of antirheumatic drugs, including conventional synthetic disease modifying anti-rheumatic drugs (csDMARDs) and biologics, have been published in 2016 [8–10]. We surveyed rheumatologists across Canada about their management of IA pregnancy to achieve objectives of identifying consensus among respondents and establishing practice patterns.

## Methods

### Survey design

The survey on rheumatologists’ management of IA pregnancy was developed, pretested, and piloted among rheumatologists, expert researchers, and specialist clinicians. The survey consisted of 23 general and specific questions focusing on the use of csDMARDs (*n* = 12; azathioprine, chloroquine, cyclophosphamide, cyclosporine, doxycycline, gold salts, hydroxychloroquine, leflunomide, methotrexate, minocycline, mycophenolate mofetil, and sulfasalazine), biologics/targeted small molecules (n = 12; abatacept, adalimumab, anakinra, apremilast, certolizumab, etanercept, golimumab, infliximab, rituximab, tocilizumab, tofacitinib, and ustekinumab), non-steroidal anti-inflammatory drugs (NSAIDs; celecoxib, ibuprofen, and naproxen) and prednisone. For questions on respondents’ general knowledge of medication safety, a 3-point Likert scale was used (“safe throughout pregnancy”, “safe during certain trimesters”, “not safe at all”) along with a “not sure” option. Specific questions assessed respondents’ treatments of planned and unplanned pregnancies; for the latter, a scenario of an IA patient with a 6-week unplanned pregnancy was employed. Multiple choice options captured how long before conception or when during pregnancy a rheumatologist would discontinue a medication in planned pregnancies. Multiple choice options were also used to capture what respondents would do in unplanned pregnancies (“continue drug, continue pregnancy”, “stop drug, continue pregnancy”, “continue drug, counsel regarding termination”, “stop drug, counsel regarding termination”, and “not sure”). The complete survey is in Additional file 1.

### Survey administration and analysis

Members of the Canadian Rheumatology Association (CRA), including rheumatologists and rheumatology trainees, were invited to participate in the survey, available in English and French and hosted online using Fluid Surveys (Ottawa, Ontario). The CRA sent unique email invitations describing the purpose of the survey and number of questions, to 450 members with two reminders in March 2016 (Additional file 2). Respondents provided consent prior to survey commencement. Anonymized responses were exported after survey completion and kept for 5 years. We used descriptive statistics, namely calculated counts and frequencies of survey responses, and a priori set cut-off of > 75% respondents to define consensus. With no prior survey studies to model defining consensus, we drew from a 2014 systematic review describing how consensus is operationalized in Delphi studies, which reported the most common definition was percent agreement, with 75% being the median threshold [11].

This study was reviewed and approved by the University of British Columbia Behavioural Research Ethics Board (#H14–02123).

## Results

All together 96 CRA members accessed the survey, 90 consented to participate (20% participation rate), and 68 provided responses to all questions (76% completion rate); nonetheless, all responses were analyzed. Table 1 summarizes respondent characteristics. Over half of respondents (57%) have been practicing rheumatology for over 10 years, 32% for less than 10 years, and 11% were trainees. The majority of respondents worked in an academic/teaching hospital setting (69%) and spent at least half of their time in direct patient care with IA patients (51%). Finally, 43% and 8% of respondents reported that the majority (26–50% and over 50%, respectively) of their IA patients were women of childbearing years, and 87% of respondents reported they continue to care these patients for during pregnancy.

**Table 1:** Characteristics of rheumatologists who completed the survey

Characteristics	*N* (%)^a^
Sex (*n* = 87)	
Female	50 (58)
Male	37 (43)
Province (n = 87)	
Ontario	27 (31)
Alberta	21 (24)
Quebec	14 (16)
British Columbia	11 (13)
Nova Scotia	4 (5)
Saskatchewan	3 (3)
Manitoba	3 (3)
New Brunswick	3 (3)
Newfoundland/Labrador	1 (1)
Practice setting (*n* = 88)	
Academic/teaching hospital	61 (69)
Group community practice	13 (15)
Solo community practice	9 (10)
Other, specify^b^	5 (6)
Percent of time spent seeing patients (n = 87)	
< 25%	11 (13)
25 to 50%	15 (17)
51 to 75%	24 (28)
> 75%	37 (43)
Years spent practicing rheumatology (n = 87)	
Currently in training	10 (11)
5 years or less	17 (19)
6 to 10 years	11 (13)
11 to 20 years	17 (20)
> 20 years	32 (37)
Proportion of patients with inflammatory arthritis (*n* = 86)	
< 25%	2 (2)
25 to 50%	15 (17)
51 to 75%	49 (57)
> 75%	20 (23)
Proportion of inflammatory arthritis patients that are women of childbearing age (n = 87)	
0	0 (0)
1 to 25%	43 (49)
26 to 50%	37 (43)
51 to 75%	5 (6)
> 75%	2 (2)
Refer pregnant inflammatory arthritis patients or those considering pregnancy to an “expert” colleague (n = 87)	
Yes	33 (38)
No	54 (62)
Follow inflammatory arthritis patients during pregnancy (n = 87)	
Yes	76 (87)
No	11 (12)

^a^ % calculated on completed responses; ^b^ Others included: rheumatology trainee, not practicing, community with academic and research ‘agenda’, subspecialized academic clinic, and mixed academic/solo community practice

### Conventional synthetic DMARDs

**Fig. 1:**
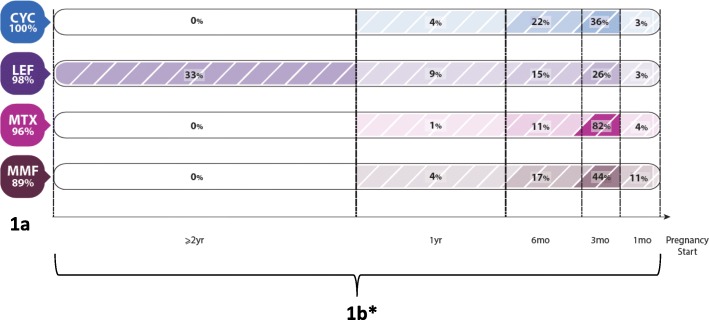
**a** Consensus on csDMARDs respondents stop in IA patients planning pregnancy (question 15) and (**b**) Responses to question on how long before conception they are stopped (question 16) (*numbers do not add to 100% as other response options are not on a time-scale [e.g. ‘do not stop’, ‘not sure’]). Abbreviations: CYC – cyclophosphamide; LEF – leflunomide; MTX – methotrexate; and MMF – mycophenolate mofetil

When queried on their general knowledge of medications safety during pregnancy (question 11), respondents achieved consensus on 4 csDMARDs that were considered not safe at all: leflunomide (98%), methotrexate (98%), cyclophosphamide (95%), and mycophenolate mofetil (82%) (Table 2). Regarding which csDMARDs respondents stop for IA patients planning pregnancy (question 15), consensus was achieved for the same 4: cyclophosphamide (100%), leflunomide (98%), methotrexate (96%), and mycophenolate mofetil (89%) (Fig. 1). The timescale in Fig. 1illustrates responses to how long before conception these 4 csDMARDs are discontinued (question 16). There was consensus among 82% of respondents on stopping methotrexate at least 3 months before pregnancy. Further, the majority of respondents indicated cyclophosphamide and mycophenolate mofetil should be discontinued at least 3 months before pregnancy; however, responses for timing leflunomide discontinuation were varied, which may reflect variability regarding the use of a cholesteryamine washout.

**Table 2:** Respondents’ general knowledge on the safety of conventional synthetic disease modifying anti-rheumatic drugs, biologics/small molecules, and other medications in the management of inflammatory arthritis in pregnancy

	Safe throughout pregnancy (% of respondents)	Safe during certain trimesters (% of respondents)	Not safe at all (% of respondents)	Not sure (% of respondents)
Conventional synthetic disease modifying anti-rheumatic drugs				
Azathioprine	80.5*	4.9	12.2	2.4
Chloroquine	74.1	1.2	8.6	16.0
Cyclophosphamide	0	3.7	95.1*	1.2
Cyclosporine	28.0	6.1	42.7	23.2
Doxycycline	2.4	4.9	59.8	32.9
Gold salts	28.7	2.5	33.8	35.0
Hydroxychloroquine	92.7*	4.9	1.2	1.2
Leflunomide	0	0	97.6*	2.4
Methotrexate	0	1.2	97.6*	1.2
Minocycline	0	4.9	53.1	42.0
Mycophenolate mofetil	2.5	2.5	81.5*	13.6
Sulfasalazine	70.4	8.6	18.5	2.5
Biologics/small molecules				
Abatacept	9.8	9.8	15.9	64.6
Adalimumab	37.0	32.1	6.2	24.7
Anakinra	9.9	6.2	16.0	67.9
Apremilast	0	1.2	18.5	80.2*
Certolizumab	61.0	15.9	4.9	18.3
Etanercept	43.9	29.3	7.3	19.5
Golimumab	37.8	25.6	11.0	25.6
Infliximab	37.8	30.5	8.5	23.2
Rituximab	8.5	8.5	25.6	57.3
Tocilizumab	11.0	8.5	19.5	61.0
Tofacitinib	1.2	1.2	21.0	76.5*
Ustekinumab	4.9	6.1	18.3	70.7
Other medications				
Celecoxib	0	54.9	29.3	15.9
Ibuprofen	1.2	83.6*	14.5	1.2
Naproxen	2.4	82.9*	11.0	3.7
Other NSAIDs	1.2	67.5	22.5	8.8

*Indicates consensus among respondents based on a priori cut-off of ≥75%

**Fig. 2:**
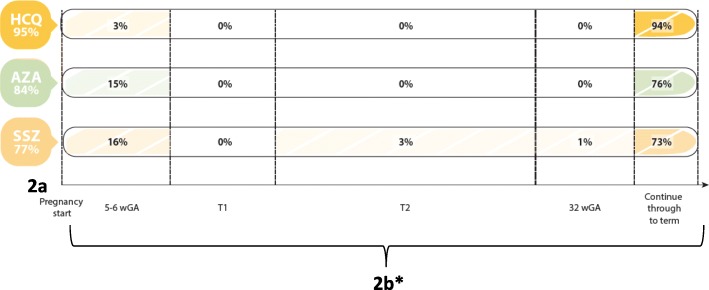
**a** Consensus on csDMARDs respondents continue in IA patients planning pregnancy (question 15) and (**b**) Responses to question on when during pregnancy they are stopped (question 17) (*numbers do not add to 100% as other response options are not on a time-scale [e.g. ‘continue do not stop’, ‘not sure’]). Abbreviations: HCQ – hydroxychloroquine; AZA – azathioprine; SSZ – sulfasalazine

Two csDMARDs were agreed to be safe during pregnancy, hydroxychloroquine (93%) and azathioprine (81%), based on responses to general knowledge of medication safety (question 11). Near consensus was achieved with chloroquine and sulfasalazine with 74% and 70% of respondents, respectively, considering them safe (Table 2). Regarding medications respondents continue during planned pregnancies (question 15), there was consensus with hydroxychloroquine (95%) azathioprine (84%), and sulfasalazine (77%) (Fig. 2). Finally, concerning when during pregnancy these csDMARDs are discontinued (question 16), respondents agreed hydoxychloroquine (94%), azathioprine (76%), and sulfasalazine (73%) can be continued throughout pregnancy (Fig. 2).

Finally, Table 3 summarizes responses for managing IA in patients with a 6-week unplanned pregnancy. Variability in survey responses suggest limited consensus; particularly, no consensus was reached regarding which csDMARDs respondents would stop. Agreement was achieved for 3 csDMARDs that respondents would continue during an unplanned pregnancy – hydroxychloroquine (91%), azathioprine (80%), and sulfasalazine (77%).

**Table 3:** Responses on the management of the inflammatory arthritis in patient with a 6-week unplanned pregnancy with conventional synthetic disease modifying anti-rheumatic drugs, biologics/small molecules, and other medications

	Continue drug, continue pregnancy (%)	Stop drug, continue pregnancy (%)	Continue drug, counsel regarding termination (%)	Stop drug, counsel regarding termination (%)	Not sure (%)
Conventional synthetic disease modifying anti-rheumatic drugs					
Azathioprine	80.3*	6.1	1.5	4.5	7.6
Chloroquine	71.2	7.6	3.0	0	18.2
Cyclophosphamide	0	16.7	13.6	60.6	9.1
Cyclosporine	27.3	18.2	1.5	19.7	33.3
Doxycycline	0	29.2	0	6.2	64.6
Gold salts	30.3	18.2	0	9.1	42.4
Hydroxychloroquine	90.9*	4.5	1.5	0	3.0
Leflunomide	0	16.7	6.1	69.7	7.6
Methotrexate	0	22.7	7.6	65.2	4.5
Minocycline	0	34.8	0	6.1	69.7
Mycophenolate mofetil	3.0	19.7	10.6	47.0	19.7
Sulfasalazine	76.6*	14.1	0	1.6	7.8
Biologics/small molecules					
Abatacept	24.2	45.5	1.5	6.1	22.7
Adalimumab	66.7	25.8	1.5	1.5	4.5
Anakinra	15.2	39.4	1.5	4.5	39.4
Apremilast	4.6	35.4	0	6.2	53.8
Certolizumab	63.1	26.2	1.5	0	9.2
Etanercept	69.7	24.2	0	1.5	4.5
Golimumab	60.6	28.8	1.5	1.5	7.6
Infliximab	60.0	29.2	1.5	1.5	7.7
Rituximab	18.2	39.4	1.5	6.1	34.8
Tocilizumab	18.2	39.4	1.5	4.5	36.4
Tofacitinib	3.0	31.8	0	7.6	57.6
Ustekinumab	13.8	33.8	1.5	4.6	46.2
Other medications					
Celecoxib	39	56	0	0	3
Ibuprofen	64	33	0	0	3
Naproxen	65	32	0	0	3
Prednisone	85*	12	0	0	3

*Indicates consensus among respondents based on a priori cut-off of ≥75%

### Biologics/small molecules

**Fig. 3:**
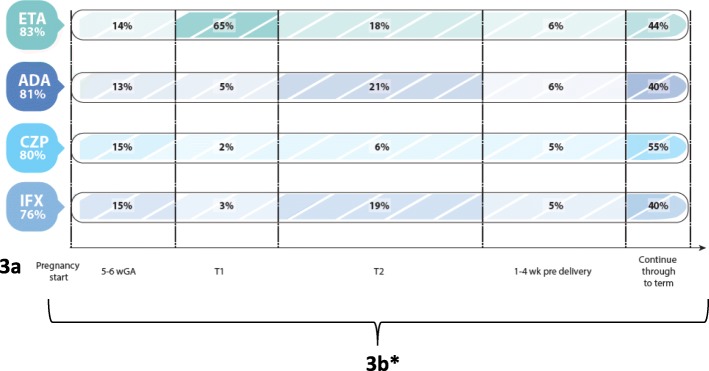
**a** Consensus on biologics respondents continue in IA patients planning pregnancy (question 18) and (**b**) Responses to question on when during pregnancy they are stopped (question 20) (*numbers do not add to 100% as other response options are not on a time-scale [e.g. ‘do not stop’, ‘not sure’]). Abbreviations: ETA – etanercept; ADA – adalimumab; CZP – certolizumab; IFX – infliximab

Responses for general knowledge of biologics/small molecule safety during pregnancy suggest uncertainty (question 12); the only consensus achieved was 80% and 77% of respondents, respectively, indicating being unsure about apremilast and tofacitinib safety, reflecting the recentness of their introduction into the market (Table 2). In contrast, when asked questions regarding biologic/small molecules use in IA patients planning pregnancy (question 18), we observed consensus for continuation of 4: etanercept (83%), adalimumab (81%), certolizumab (80%), and infliximab (77%) (Fig. 3). However, there was response variability regarding when during planned pregnancy rheumatologists would discontinue these biologics (Fig. 3). Finally, the summarized responses for managing IA in patients with a 6-week unplanned pregnancy (Table 3) show no consensus on biologics/small molecules use.

### Other medications

When asked about general knowledge of other medications safety during pregnancy, 84 and 83% agreed that ibuprofen and naproxen, respectively, were safe during certain trimesters (Table 2). However, when asked specific questions about medications that respondents stopped in IA patients planning pregnancy, no consensus was observed with NSAIDs. Conversely, 91% of respondents indicated they do not stop prednisone in planned pregnancies. In specific questions regarding unplanned pregnancies, there was no consensus regarding NSAIDs but 85% of respondents agreed that they would continue prednisone and pregnancy (Table 3).

### Guidance needed by respondents

The final question asked respondents to indicate issues missed by survey questions or other areas regarding IA treatment during pregnancy that they wish further guidance on. Overall, 32 (36%) respondents provided unique responses to this section and indicated that they needed more nuanced guidance for patient-specific considerations when making decisions concerning medication management and pregnancy in female IA patients. They listed disease type and severity, availability of high-resolution ultrasound for monitoring fetal development, gestational age, delivery type, continued medication use, and plan to breastfeed as considerations for making changes in disease management and counselling on pregnancy progression. Respondents were interested in the impact of medications used in male partners on pregnancy outcomes. Given the prevalence of anti-inflammatory medication use in IA, respondents hoped for more guidance on considerations for NSAID and prednisone use throughout pregnancy if needed for disease management and for restarting medications post-pregnancy. Respondents mentioned patient non-adherence as a barrier to disease and pregnancy management. They further stressed the need for shared decision making with patients when counselling on the risks of pregnancy continuation.

## Discussion

This national rheumatologist survey focused on knowledge of medication safety, primarily csDMARDs and biologics/small molecules, and respondents’ practice in planned and unplanned pregnancies. There was consensus on discontinuation of 4 csDMARDs (cyclophosphamide, leflunomide, methorexate, and mycophenolate mofetil) in planned pregnancies but limited knowledge on when to stop them prior to pregnancy and no consensus for unplanned pregnancies. Respondents agreed that 3 csDMARDs (azathioprine, hydroxychloroquine, and sulfasalazine) were safe in all pregnancies. There was consensus for using 4 biologics/small molecules (adalimumab, etanercept, certolizumab, and infliximab) in planned pregnancies but uncertainty on when to discontinue them and no consensus in unplanned pregnancies. There was consensus with prednisone use in planned and unplanned pregnancies but uncertainty regarding NSAIDs.

Our survey informs rheumatologists’ practice patterns for IA treatment in pregnancy with implications for identifying gaps in knowledge and research. Guidance for the use of antirheumatic drugs before and during pregnancy from the European League Against Rheumatism (EULAR) [8] and from the British Society for Rheumatology (BSR) and British Health Professionals in Rheumatology (BHPR) on prescribing csDMARDs and biologics in pregnancy [9], were both published in 2016, at the time of our survey administration. Consequently, survey responses may reflect understanding of these guidelines or, given the brief period between their publication and our survey’s administration, baseline practice patterns for future evaluation of the impacts of these guidelines. Nonetheless, survey responses align with EULAR points for discontinuation of methotrexate, cyclophosphamide, and mycophenolate mofetil before pregnancy and continuation of antimalarials, azathioprine, and sulfasalazine. Regarding biologics/small molecules, the EULAR guidance support use of anti-TNFs during the start of pregnancy, with certolizumab and etanercept use being acceptable throughout pregnancy as they have little transplacental passage. While survey responses regarding biologics/small molecules continuation in planned pregnancies largely aligned with these guidelines, medication discontinuation during pregnancy is not fully addressed by the EULAR points. Furthermore, the EULAR points do not address actions in unplanned pregnancies for which we observed uncertainty for 9 of 12 csDMARDs and all 12 biologics/small molecules queried.

The BSR BHPR guidelines address gaps in the EULAR points, particularly when medications should be discontinued. For csDMARDs incompatible with pregnancy, cyclosporine should not be used peri-conception, methotrexate should be stopped 3 months before conception, and mycophenolate mofetil 6 weeks in advance [9]. Leflunomide was suggested to only be compatible during peri-conception if accompanied by a cholestyramine washout regimen [9]. Survey responses for the discontinuation of methotrexate aligned with these generally more conservative guidelines. Further, responses on azathioprine, hydroxychloroquine and sulfasalazine use align with the BSR BHPR guidelines for their continuation during pregnancy. Among TNF-alpha inhibitor biologics, only certolizumab was recommended for use throughout pregnancy, golimumab had no data to support any recommendations, and all others were recommended for first trimester (infliximab) or first and second trimester use only (etanercept and adalimumab) [9]. The BSR BHPR guidelines acknowledged the lack of evidence to support recommendations for non-TNF alpha inhibitor biologics/small molecules but stated unintentional first trimester exposure to these drugs would unlikely be harmful [9]. While these guidelines address gaps observed in our survey for biologics/small molecules use in planned pregnancies, the BSR BHPR guidelines largely do not address medication use in unplanned pregnancies, only touching on methotrexate and leflunomide in ‘accidental’ pregnancies. Given that up to 50% of pregnancies are unplanned [12], our survey points to potential areas needing attention in future guideline work.

Multiple factors may explain the variability of survey responses. When treating maternal illness in pregnancy, disease severity and potential disease effect on pregnancy outcomes need to be balanced against potential treatment risks to developing fetus. Active IA, particularly RA, psoriatic arthritis and ankylosing spondylitis, can damage joints and lead to maternal disability if untreated [13, 14]. Furthermore, active IA, particularly RA, can have adverse effects on pregnancy outcome (small for gestational age infants and premature delivery) [13, 14]. Thus, it is important that maternal disease activity be controlled before and during pregnancy with medications that are safe for the fetus. If maternal disease is in remission or has low disease activity, no or little treatment may be required. Each rheumatologist and patient must decide together the severity of maternal disease activity and balance it against the risk of adverse pregnancy outcomes and potential fetal harm. Patient comfort levels taking medication during pregnancy also needs addressing. Additionally, response variability may be due to relative lack of data regarding medication use in pregnancy compared to the non-pregnant state. It is unethical to perform prospective, double-blind, randomized controlled trials of medications safety in pregnancy. There may be animal data on the effect of some drugs which may or may not be applicable to humans. As such, the only method to obtain drug data in human pregnancies is when a mother becomes pregnant while taking a medication or takes one during pregnancy and the outcomes of the pregnancy and fetus are documented. Further, to collect a sufficient number of case reports of pregnancies exposed to a particular medication requires it be on the market for many years. For new medications, there will be almost no data available on that drug in human pregnancies. Lack of data from prospective randomized trials leaves a gap in knowledge regarding the medication safety in pregnancy, especially newer medications. Some clinicians are satisfied with less observational data and others require more before prescribing drugs in pregnancy; as such, there are variable levels of comfort among prescribers. These aforementioned reasons may be more relevant to explaining survey responses for planned pregnancies. With unplanned pregnancies, such reasons apply along with considerations on values and beliefs around termination of pregnancy.

Limitations of our survey study deserve comment. First, despite centralized invitation from the CRA, the sample size was small and participation rate (20%) was modest. However, the aforementioned survey of UK rheumatologists on DMARDs use during pregnancy similarly yielded a 20% response rate [7]. Additionally, prior surveys to CRA membership using the same methods have yielded similar response rates [15, 16]. With 69% of respondents representing rheumatologists practicing in academic or teaching hospital settings, findings may be subject to potential selection bias. Despite querying 12 csDMARDs, 12 biologics/small molecules, 3 NSAIDs, and prednisone, our survey omitted some medications covered by the EULAR points (e.g. tacrolimus) and BSR BHPR (e.g. belimumab). In addition, survey scenarios with planned and unplanned pregnancies in IA patients queried did not incorporate disease activity or severity, which are important considerations. Finally, our survey did not ask rheumatologists about recommendations made to patients after delivery, specifically, regarding breastfeeding.

## Conclusion

This national survey shows consensus among rheumatologists on the safety of some csDMARDs and biologics/small molecules in IA patients planning pregnancy. However, there was limited knowledge on when to discontinue these medications and what to do in unplanned pregnancies. Findings are timely as they establish baseline practice patterns and identify gaps that may be addressed by recently published points and guidelines.

## Additional files

Additional file 1: Rheumatologist Survey on the Management of Inflammatory Arthritis in Pregnancy (PDF 210 kb)

Additional file 2: Survey invitations sent via email to Canadian Rheumatology Association members (English and in French) (PDF 49 kb)

## Abbreviations

ADA: Adalimumab
anti-TNFs: Anti-tumour necrosis factor agents
AZA: Azathioprine
BHPR: British Health Professionals in Rheumatology
BSR: British Society for Rheumatology
CRA: Canadian Rheumatology Association
csDMARDs: Conventional synthetic disease modifying antirheumatic drugs
CYC: Cyclophosphamide
CZP: Certolizumab
DMARDs: Disease modifying antirheumatic drugs
ETA: Etanercept
EULAR: European League Against Rheumatism
HCQ: Hydroxychloroquine
IA: Inflammatory arthrides
IFX: Infliximab
LEF: Leflunomide
MMF: Mycophenolate mofetil
MTX: Methotrexate
NSAIDs: Non-steroidal anti-inflammatory drugs
RA: Rheumatoid arthritis
SLE: Systemic lupus erythematosus
SSZ: Sulfasalazine
TNF: Tumour necrosis factor
UK: United Kingdom
