# Supplementary material for: Management of Inflammatory Arthritis in pregnancy: a National Cross-Sectional Survey of Canadian rheumatologists
Source: BMC Rheumatol. 2019 May 17;3:18. doi: 10.1186/s41927-019-0065-8 (PMC6530147; doi:10.1186/s41927-019-0065-8)
Supplement: Supplementary file 1 — Rheumatologist Survey on the Management of Inflammatory Arthritis in Pregnancy (PDF 210 kb) [file 41927_2019_65_MOESM1_ESM.pdf]

Additional file 1. Rheumatologist Survey on the Management of Inflammatory Arthritis in Pregnancy.*

1. **How would you describe your practice setting?**

[ ] Solo community practice

[ ] Group community practice

[ ] Clinical trials research facility associated with community practice

[ ] Academic/teaching hospital

[ ] Other, please specify...

2. **What is your gender?**

[ ] Male

[ ] Female

3. **In what province do you practice?**

[ ] AB

[ ] BC

[ ] MB

[ ] NB

[ ] NL

[ ] NT

[ ] NS

[ ] NU

[ ] ON

[ ] PE

[ ] QC

[ ] SK

[ ] YT

4. **What percentage of your time do you spend seeing patients?**

[ ] <25%

[ ] 25-50%

[ ] 51-75%

[ ] >75%

*The questionnaire was distributed over 8 screens, which included the study information and consent page. Respondents were able to review and change their answers prior to submission.

5. **How many years have you practiced rheumatology?**
   [ ] Currently in a residency or fellowship training program
   [ ] ≤1 to 5 years
   [ ] 6 to 10 years
   [ ] 11 to 20 years
   [ ] >20 years

6. **What proportion of your patients have inflammatory arthritis?**
   [ ] <25%
   [ ] 25-50%
   [ ] 51-75%
   [ ] >75%

7. **What proportion of your inflammatory arthritis patients are women of childbearing age?**
   [ ] 0%
   [ ] 1-25%
   [ ] 26-50%
   [ ] 51-75%
   [ ] >75%

8. **Do you send patients considering pregnancy or those who are pregnant to an "expert" colleague?**
   [ ] Yes
   [ ] No

9. **Do you follow your inflammatory arthritis patients during pregnancy?**
   [ ] Yes
   [ ] No

10. How safe do you think the following drugs are during pregnancy?

	Safe throughout pregnancy	Safe during certain trimesters	Not safe at all	Not sure
Ibuprofen	[ ]	[ ]	[ ]	[ ]
Naproxen	[ ]	[ ]	[ ]	[ ]
Celecoxib	[ ]	[ ]	[ ]	[ ]
Other NSAIDs	[ ]	[ ]	[ ]	[ ]

11. How safe do you think the following conventional DMARDs are during pregnancy?

	Safe throughout pregnancy	Safe during certain trimesters	Not safe at all	Not sure
Azathioprine	[ ]	[ ]	[ ]	[ ]
Chloroquine	[ ]	[ ]	[ ]	[ ]
Cyclophosphamide	[ ]	[ ]	[ ]	[ ]
Cyclosporine	[ ]	[ ]	[ ]	[ ]
Doxycycline	[ ]	[ ]	[ ]	[ ]
Gold Salts	[ ]	[ ]	[ ]	[ ]
Hydroxychloroquine	[ ]	[ ]	[ ]	[ ]
Leflunomide	[ ]	[ ]	[ ]	[ ]
Methotrexate	[ ]	[ ]	[ ]	[ ]
Minocycline	[ ]	[ ]	[ ]	[ ]
Mycophenolate mofetil	[ ]	[ ]	[ ]	[ ]
Sulfasalazine	[ ]	[ ]	[ ]	[ ]

12. How safe do you think the following biologic DMARDs/small molecules are during pregnancy?

	Safe throughout pregnancy	Safe during certain trimesters	Not safe at all	Not sure
Abatacept	[ ]	[ ]	[ ]	[ ]
Adalimumab	[ ]	[ ]	[ ]	[ ]
Anakinra	[ ]	[ ]	[ ]	[ ]
Apremilast	[ ]	[ ]	[ ]	[ ]
Certolizumab	[ ]	[ ]	[ ]	[ ]
Etanercept	[ ]	[ ]	[ ]	[ ]
Golimumab	[ ]	[ ]	[ ]	[ ]
Infliximab	[ ]	[ ]	[ ]	[ ]
Rituximab	[ ]	[ ]	[ ]	[ ]
Tocilizumab	[ ]	[ ]	[ ]	[ ]
Tofacitinib	[ ]	[ ]	[ ]	[ ]
Ustekinumab	[ ]	[ ]	[ ]	[ ]

13. Which, if any, of the following drugs do you stop in the inflammatory arthritis patient planning pregnancy?

	Stop	May continue	Not sure
Ibuprofen	[ ]	[ ]	[ ]
Naproxen	[ ]	[ ]	[ ]
Celecoxib	[ ]	[ ]	[ ]
Other NSAIDs	[ ]	[ ]	[ ]

14. Your inflammatory arthritis patient is planning pregnancy. How long before conception do you advise the discontinuation of the following drugs?

	1-2 wks	1 mo	3 mo	6 mo	Do not stop	Not sure
Ibuprofen	[ ]	[ ]	[ ]	[ ]	[ ]	[ ]
Naproxen	[ ]	[ ]	[ ]	[ ]	[ ]	[ ]
Celecoxib	[ ]	[ ]	[ ]	[ ]	[ ]	[ ]
Other NSAIDs	[ ]	[ ]	[ ]	[ ]	[ ]	[ ]
Prednisone	[ ]	[ ]	[ ]	[ ]	[ ]	[ ]

15. Which, if any, of the following conventional DMARDs do you stop in the inflammatory arthritis patient planning pregnancy?

	Stop	May continue	Not sure
Azathioprine	[ ]	[ ]	[ ]
Chloroquine	[ ]	[ ]	[ ]
Cyclophosphamide	[ ]	[ ]	[ ]
Cyclosporine	[ ]	[ ]	[ ]
Doxycycline	[ ]	[ ]	[ ]
Gold Salts	[ ]	[ ]	[ ]
Hydroxychloroquine	[ ]	[ ]	[ ]
Leflunomide	[ ]	[ ]	[ ]
Methotrexate	[ ]	[ ]	[ ]
Minocycline	[ ]	[ ]	[ ]
Mycophenolate mofetil	[ ]	[ ]	[ ]
Sulfasalazine	[ ]	[ ]	[ ]

16. Your inflammatory arthritis patient is planning pregnancy. How long before conception do you advise the discontinuation of the following conventional DMARDs?

	1-2 mo	3 mo	6 mo	1 yr	≥2 yrs	Do not stop	Pregnancy not rec'd at this	Not sure
Azathioprine	[ ]	[ ]	[ ]	[ ]	[ ]	[ ]	[ ]	[ ]
Chloroquine	[ ]	[ ]	[ ]	[ ]	[ ]	[ ]	[ ]	[ ]
Cyclophosphamide	[ ]	[ ]	[ ]	[ ]	[ ]	[ ]	[ ]	[ ]
Cyclosporine	[ ]	[ ]	[ ]	[ ]	[ ]	[ ]	[ ]	[ ]
Doxycycline	[ ]	[ ]	[ ]	[ ]	[ ]	[ ]	[ ]	[ ]
Gold Salts	[ ]	[ ]	[ ]	[ ]	[ ]	[ ]	[ ]	[ ]
Hydroxychloroquine	[ ]	[ ]	[ ]	[ ]	[ ]	[ ]	[ ]	[ ]
Leflunomide	[ ]	[ ]	[ ]	[ ]	[ ]	[ ]	[ ]	[ ]
Methotrexate	[ ]	[ ]	[ ]	[ ]	[ ]	[ ]	[ ]	[ ]
Minocycline	[ ]	[ ]	[ ]	[ ]	[ ]	[ ]	[ ]	[ ]
Mycophenolate mofetil	[ ]	[ ]	[ ]	[ ]	[ ]	[ ]	[ ]	[ ]
Sulfasalazine	[ ]	[ ]	[ ]	[ ]	[ ]	[ ]	[ ]	[ ]

17. Your inflammatory arthritis patient chooses to continue with her conventional DMARDs while attempting to conceive and is now 6 weeks pregnant. When would you advise discontinuation of the following conventional DMARDs?

	Estimated gestational age of 5-6 weeks	End of 1st trimester	End of 2nd trimester	At 32 weeks' gestation	Continue, do not stop	Counsel regarding termination	Not sure
Azathioprine	[ ]	[ ]	[ ]	[ ]	[ ]	[ ]	[ ]
Chloroquine	[ ]	[ ]	[ ]	[ ]	[ ]	[ ]	[ ]
Cyclophosphamide	[ ]	[ ]	[ ]	[ ]	[ ]	[ ]	[ ]
Cyclosporine	[ ]	[ ]	[ ]	[ ]	[ ]	[ ]	[ ]
Doxycycline	[ ]	[ ]	[ ]	[ ]	[ ]	[ ]	[ ]
Gold Salts	[ ]	[ ]	[ ]	[ ]	[ ]	[ ]	[ ]
Hydroxychloroquine	[ ]	[ ]	[ ]	[ ]	[ ]	[ ]	[ ]
Leflunomide	[ ]	[ ]	[ ]	[ ]	[ ]	[ ]	[ ]
Methotrexate	[ ]	[ ]	[ ]	[ ]	[ ]	[ ]	[ ]
Minocycline	[ ]	[ ]	[ ]	[ ]	[ ]	[ ]	[ ]
Mycophenolate mofetil	[ ]	[ ]	[ ]	[ ]	[ ]	[ ]	[ ]
Sulfasalazine	[ ]	[ ]	[ ]	[ ]	[ ]	[ ]	[ ]

18. Which, if any, of the following biologic DMARDs/small molecules do you stop in the inflammatory arthritis patient planning pregnancy?

	Stop	May continue	Not sure
Abatacept	[ ]	[ ]	[ ]
Adalimumab	[ ]	[ ]	[ ]
Anakinra	[ ]	[ ]	[ ]
Apremilast	[ ]	[ ]	[ ]
Certolizumab	[ ]	[ ]	[ ]
Etanercept	[ ]	[ ]	[ ]
Golimumab	[ ]	[ ]	[ ]
Infliximab	[ ]	[ ]	[ ]
Rituximab	[ ]	[ ]	[ ]
Tocilizumab	[ ]	[ ]	[ ]
Tofacitinib	[ ]	[ ]	[ ]
Ustekinumab	[ ]	[ ]	[ ]

19. Your inflammatory arthritis patient is planning pregnancy. How long before conception do you advise the discontinuation of the following biologic DMARDs/small molecules?

	1 wks	2 wks	4 wks	8 wks	12 wks	6 mo	1 yr	Do not stop	Not sure
Abatacept	[ ]	[ ]	[ ]	[ ]	[ ]	[ ]	[ ]	[ ]	[ ]
Adalimumab	[ ]	[ ]	[ ]	[ ]	[ ]	[ ]	[ ]	[ ]	[ ]
Anakinra	[ ]	[ ]	[ ]	[ ]	[ ]	[ ]	[ ]	[ ]	[ ]
Apremilast	[ ]	[ ]	[ ]	[ ]	[ ]	[ ]	[ ]	[ ]	[ ]
Certolizumab	[ ]	[ ]	[ ]	[ ]	[ ]	[ ]	[ ]	[ ]	[ ]
Etanercept	[ ]	[ ]	[ ]	[ ]	[ ]	[ ]	[ ]	[ ]	[ ]
Golimumab	[ ]	[ ]	[ ]	[ ]	[ ]	[ ]	[ ]	[ ]	[ ]
Infliximab	[ ]	[ ]	[ ]	[ ]	[ ]	[ ]	[ ]	[ ]	[ ]
Rituximab	[ ]	[ ]	[ ]	[ ]	[ ]	[ ]	[ ]	[ ]	[ ]
Tocilizumab	[ ]	[ ]	[ ]	[ ]	[ ]	[ ]	[ ]	[ ]	[ ]
Tofacitinib	[ ]	[ ]	[ ]	[ ]	[ ]	[ ]	[ ]	[ ]	[ ]
Ustekinumab	[ ]	[ ]	[ ]	[ ]	[ ]	[ ]	[ ]	[ ]	[ ]

20. Your inflammatory arthritis patient chooses to continue with her biologic DMARDs and small molecules while attempting to conceive and is now 6 weeks pregnant. When would you advise discontinuation of the following biologic DMARDs/small molecules?

	Estimated gestational age of 5-6 weeks	End 1st trimester	End 2nd trimester	1-4 wks prior to delivery	Do not stop	Disagree: stop before conception	Not sure
Abatacept	[ ]	[ ]	[ ]	[ ]	[ ]	[ ]	[ ]
Adalimumab	[ ]	[ ]	[ ]	[ ]	[ ]	[ ]	[ ]
Anakinra	[ ]	[ ]	[ ]	[ ]	[ ]	[ ]	[ ]
Apremilast	[ ]	[ ]	[ ]	[ ]	[ ]	[ ]	[ ]
Certolizumab	[ ]	[ ]	[ ]	[ ]	[ ]	[ ]	[ ]
Etanercept	[ ]	[ ]	[ ]	[ ]	[ ]	[ ]	[ ]
Golimumab	[ ]	[ ]	[ ]	[ ]	[ ]	[ ]	[ ]
Infliximab	[ ]	[ ]	[ ]	[ ]	[ ]	[ ]	[ ]
Rituximab	[ ]	[ ]	[ ]	[ ]	[ ]	[ ]	[ ]
Tocilizumab	[ ]	[ ]	[ ]	[ ]	[ ]	[ ]	[ ]
Tofacitinib	[ ]	[ ]	[ ]	[ ]	[ ]	[ ]	[ ]
Ustekinumab	[ ]	[ ]	[ ]	[ ]	[ ]	[ ]	[ ]

21. Your inflammatory arthritis patient has a 6 week unplanned pregnancy and is still taking medications. How should the patient proceed?

	Continue drug, continue pregnancy	Stop drug, continue pregnancy	Continue drug, counsel regarding termination	Stop drug, counsel regarding termination	Not sure
Ibuprofen	[ ]	[ ]	[ ]	[ ]	[ ]
Naproxen	[ ]	[ ]	[ ]	[ ]	[ ]
Celecoxib	[ ]	[ ]	[ ]	[ ]	[ ]
Other NSAIDs	[ ]	[ ]	[ ]	[ ]	[ ]
Prednisone	[ ]	[ ]	[ ]	[ ]	[ ]

22. Your inflammatory arthritis patient has a 6 week unplanned pregnancy and is still taking her conventional DMARD. How should the patient proceed?

	Continue drug, continue pregnancy	Stop drug, continue pregnancy	Continue drug, counsel regarding termination	Stop drug, counsel regarding termination	Not sure
Azathioprine	[ ]	[ ]	[ ]	[ ]	[ ]
Chloroquine	[ ]	[ ]	[ ]	[ ]	[ ]
Cyclophosphamide	[ ]	[ ]	[ ]	[ ]	[ ]
Cyclosporine	[ ]	[ ]	[ ]	[ ]	[ ]
Doxycycline	[ ]	[ ]	[ ]	[ ]	[ ]
Gold Salts	[ ]	[ ]	[ ]	[ ]	[ ]
Hydroxychloroquine	[ ]	[ ]	[ ]	[ ]	[ ]
Leflunomide	[ ]	[ ]	[ ]	[ ]	[ ]
Methotrexate	[ ]	[ ]	[ ]	[ ]	[ ]
Minocycline	[ ]	[ ]	[ ]	[ ]	[ ]
Mycophenolate mofetil	[ ]	[ ]	[ ]	[ ]	[ ]
Sulfasalazine	[ ]	[ ]	[ ]	[ ]	[ ]

23. Your inflammatory arthritis patient has a 6 week unplanned pregnancy and is still taking her biologic DMARD/small molecule. How should the patient proceed?

	Continue drug, continue pregnancy	Stop drug, continue pregnancy	Continue drug, counsel regarding termination	Stop drug, counsel regarding termination	Not sure
Abatacept	[ ]	[ ]	[ ]	[ ]	[ ]
Adalimumab	[ ]	[ ]	[ ]	[ ]	[ ]
Anakinra	[ ]	[ ]	[ ]	[ ]	[ ]
Apremilast	[ ]	[ ]	[ ]	[ ]	[ ]
Certolizumab	[ ]	[ ]	[ ]	[ ]	[ ]
Etanercept	[ ]	[ ]	[ ]	[ ]	[ ]
Golimumab	[ ]	[ ]	[ ]	[ ]	[ ]
Infliximab	[ ]	[ ]	[ ]	[ ]	[ ]
Rituximab	[ ]	[ ]	[ ]	[ ]	[ ]
Tocilizumab	[ ]	[ ]	[ ]	[ ]	[ ]
Tofacitinib	[ ]	[ ]	[ ]	[ ]	[ ]
Ustekinumab	[ ]	[ ]	[ ]	[ ]	[ ]

24. What questions or issues about treating inflammatory arthritis patients in pregnancy have we missed or would you wish further guidance on?
