# Supplementary material for: Management of Inflammatory Arthritis in pregnancy: a National Cross-Sectional Survey of Canadian rheumatologists
Source: BMC Rheumatol. 2019 May 17;3:18. doi: 10.1186/s41927-019-0065-8 (PMC6530147; doi:10.1186/s41927-019-0065-8)
Supplement: Supplementary file 2 — Survey invitations sent via email to Canadian Rheumatology Association members (English and in French) (PDF 49 kb) [file 41927_2019_65_MOESM2_ESM.pdf]

Additional file 2. Survey invitations sent via email to Canadian Rheumatology Association members (English and in French).

Subject: Management of Inflammatory Arthritis in Pregnancy

Dear Dr. ___________________________:

Please help inform the development of much-needed clinical practice guidelines for the management of inflammatory arthritis during pregnancy by completing this survey.

This survey consists of 24 questions regarding your current practice when treating women with inflammatory arthritis during pregnancy. The results of this survey, along with the results of a systematic review of the literature, will be used to inform the development of Canadian clinical practice guidelines for the management of inflammatory arthritis during pregnancy.

Please click on the link below to access the survey. Thank you for your participation!

Sincerely,

Drs. Stephanie Ensworth and Mary De Vera

Sujet : Prise en charge de l'arthrite inflammatoire au cours de la grossesse

Cher / chère Dr. ______________________ :

En répondant à cette enquête, vous aidez à informer du besoin de lignes directrices de pratique clinique pour la prise en charge de l'arthrite inflammatoire au cours de la grossesse.

Ce sondage comprend 24 questions portant sur votre pratique actuelle concernant le traitement des femmes atteintes d'arthrite inflammatoire lorsqu'elles sont enceintes. Les résultats obtenus grâce à cette enquête, combinés à ceux issus d'une revue systématique de la littérature scientifique, seront utilisés pour informer du besoin d'élaborer des lignes directrices canadiennes de pratique clinique pour la prise en charge de l'arthrite inflammatoire au cours de la grossesse.

Merci de cliquer sur le lien ci-dessous pour accéder au sondage. Nous vous remercions pour votre participation!

Salutations,

Dr. Stéphanie Ensworth et Dr. Mary De Vera
